# Social Network Changes in Cotton-Top Tamarins (*Saguinus oedipus*) after the Birth of New Infants

**DOI:** 10.3390/ani13111758

**Published:** 2023-05-25

**Authors:** Sergio Díaz, Susana Sánchez, Ana Fidalgo

**Affiliations:** Departamento de Psicología Biológica y de la Salud, Universidad Autónoma de Madrid, 28049 Madrid, Spain

**Keywords:** social networks, cooperative breeding, *Saguinus oedipus*, group stability, centrality

## Abstract

**Simple Summary:**

We investigated the social structure of two groups of cooperatively breeding cotton-top tamarins (*Saguinus oedipus*) before and after the birth of infants. The two groups had different sizes and age compositions: one being smaller and only having two adults, the breeding pair, and the other being bigger and having several non-reproductive adults. We investigated the group structure by observing grooming relationships, and we studied infant care by observing the time that group members spent carrying infants. The results showed that the group with non-reproductive adults showed a stable structure after the birth of the infants, while the smaller group showed changes in their structure. We also found no evidence of a relationship between the time spent carrying infants and group position. In conclusion, the presence of non-reproductive adults could be a determinant factor in maintaining group stability, although group members do not seem to improve their social position through infant carrying.

**Abstract:**

Cotton-top tamarins (*Saguinus oedipus*) are characterized by a system of cooperative breeding where helpers, in addition to the reproductive pair, contribute to infant care. Grooming interactions between individuals play an important role in establishing social relationships, creating an interconnected social network in the group. We used social network analysis to investigate the social structure of two groups of cotton-top tamarins with different sizes and compositions and study whether they remain stable after the birth of new infants. We also investigated the possible correlation between the time spent carrying infants and an increase in the grooming centrality. We found that group A (*n* = 13) had a stable grooming network that showed consistent stability after the birth, although group B (*n* = 8 and no adult helpers) changed its grooming network and showed a lower density after the birth. Infant carrying was not correlated with increased grooming centrality after the birth. These findings highlight the usefulness of social network analysis in the study of group structure in cooperatively breeding primates and suggest that the birth of offspring has a greater impact on the stability of groups without adult helpers.

## 1. Introduction

In most primate species, infant care is a task that is commonly carried out by the mother. In addition to lactation, mothers often perform other forms of infant care that are highly important, such as carrying the infant on their bodies [1]. However, unlike nursing, infant carrying can be performed not only by the mother of the infant, but also by other individuals, resulting in cooperative systems of infant care. The most common topics of study in cooperative infant care focus on the role of the father [2,3,4] and the mother [5,6]. Cooperative infant care is an important endeavor in a group and affects family dynamics not only between the parents and the infants, but also between non-breeding group members [7]. Research investigating social dynamics in cooperatively breeding primates has also investigated adult–infant relationships [8] and competition between helpers [9]. However, less is known about how the birth of new infants can potentially affect social relationships in a cooperative primate group.

Cooperative breeding systems in primates have often been studied by examining dyads, such as father–mother or parent–helper dyads [7]. In recent years, other areas of animal and primate research have begun applying social network analysis (SNA) as an approach to investigate the social structure of groups as a whole [10,11,12]. Rather than looking into individual behaviors or into specific dyads, this approach focuses on all the relationships, known as “ties”, between any possible individual, known as “nodes”, in the group. In other words, SNA treats all data as dyadic interactions and offers statistical tools to test hypotheses at the dyadic and group levels [13]. While it is not a new approach in areas such as psychology and sociology, it only started becoming common in the study of animal behavior around the late 2000s, where primatology became one of the leading fields in utilizing SNA [14]. Reviews and issues dedicated to available tools within SNA, their potential future applications and important methodological considerations [15,16,17,18,19,20] paved the way for new and exciting research. SNA has commonly been used to inform the management of captive animals [12,21,22], including primates [23,24], due to its usefulness in studying how different social, environmental or individual factors affect group structure.

Grooming is a particularly well-studied behavior within the field of social networks, given its distinct role in affective communication [25] in primates, including in platyrrhines [26]. Grooming networks are particularly important due to the valuable social function that they provide, maintaining affiliative bonds [27,28,29] and group cohesion [30]. Grooming networks provide good metrics and indicators of individual roles and overall group cohesion [31]. An important feature of SNA is the possibility of calculating individuals’ centrality metrics. For example, a commonly used measure of centrality is the eigenvector, which indicates which individuals are well-connected with partners that are well-connected themselves [10]. This metric has been applied to proximity networks [32], affiliative networks [33] and grooming networks [31,34]. The eigenvector is particularly important when studying grooming social networks because it can be interpreted as a measure of “social support” or “social capital” [35]. Past studies investigating infant carrying and individual sociality in cooperatively breeding primates have focused on analyzing specific dyads [8,36]. Centrality measures such as the eigenvector allow new studies to analyze the overall role of group members instead of focusing on breeder–helper dyads. Thus, the eigenvector can be a useful metric to examine if the contribution to infant carrying leads to an increase in social support.

Grooming networks have proven to be useful when investigating changes in a group in different contexts or after changes to the group [37,38,39,40]. A useful measure when investigating changes in group cohesion is density, which refers to the ratio between existing links and all potential links in a network [35]. Changes in the environment such as the season [41] or space availability [34] can lead to changes in the grooming density. Additionally, developments in inferential tests developed to work with network data allow researchers to test whether the structure of the group itself is stable over time or affected by individual attributes such as age or sex [38,42]. Thanks to these tools, it is possible to analyze the impact that the birth of new infants can have on the structure of grooming networks in cooperatively breeding primates.

While most platyrrhines spend less time grooming than other primates [43], social grooming is a very prominent behavior among callitrichids, which spend a considerable amount of time engaged in this activity [44,45,46,47,48]. Callitrichids live in groups of 3 to 15 individuals in the wild, composed mainly of related individuals [49]. There is usually only one reproductive female who has a high reproductive rate owing to twin births as well as postpartum ovulations occurring 10 to 20 days after birth [50,51], and who requires help from all family members in infant care [7]. Cooperative infant care in callitrichids occurs especially through carrying and food sharing, but also through grooming, protecting and other types of care [7]. Authors have suggested that grooming plays an important role in maintaining relationships and incentivizes infant care, ensuring that some individuals remain in the group [46,47]. Interestingly, one commonly proposed incentive for infant care in callitrichines is the improvement of social prestige or popularity in the group [52,53]. If contributing to infant care leads to an increase in popularity, then it is worth studying the overall position of individuals in the grooming network, rather than focusing on parent–helper dyads.

In captive common marmosets (*Callithrix jacchus*), strong grooming relationships between breeders and helpers are positively linked to infant carrying and food sharing [36]. Research in captive common marmosets has often focused on studying dyadic relationships between breeders and helpers, although strong bonds occur not only between breeding partners, but also between all individuals [54], and, importantly, these grooming interactions are known to be stable after the birth of new infants [55]. Additionally, in captive cotton-top tamarins (*Saguinus oedipus*), grooming plays an important role in the group, as grooming received by helpers is associated with infant carrying [56]. Since tamarins are thought to have the highest costs associated with infant care among callitrichines [57], increased social popularity is to be expected in those individuals that invest in helping. Eigenvector centrality can further advance this topic of research, as it allows researchers to clearly examine if contributions to infant carrying correlate with an increased popularity in the group.

Cotton-top tamarins are particularly relevant to this topic as their social system is characterized by shared infant care [58] and there is an extensive body of research in this species that could be further advanced using SNA. Cotton-top tamarins typically live in groups of three to ten members, formed by one reproductive male, one reproductive female and one or more other members, often the offspring of the reproductive members [59,60]. Infant carrying is a crucial aspect of infant care as the offspring typically do not begin to show independent locomotion until after the first month of life [58]. In the wild, adults carry infants continuously until their seventh week of life [60]. While the mother plays an important role in infant care [6], the reproductive male of the group offers a big contribution to infant carrying and caring, often suffering important weight loss as a consequence [2,3,4]. Expectant fathers gain weight during their mate’s pregnancy related to hormonal changes occurring to prepare them for the subsequent body mass loss [61,62,63]. Despite these costs, fathers and helpers often compete in order to carry the infants [64,65]. Groups with multiple adult helpers reduce the costs of father-to-infant care, as helpers significantly contribute to infant care, carrying infants and sharing food with them [66]. Helpers are a crucial component of infant care and can determine the survival chance of the infants [67]. The age of the helpers is an important factor to consider, as adult and subadult helpers spend more time carrying infants than juvenile helpers [8]. Older helpers also have more experience and skill in infant care, similar to how older parents show more infant-caring behaviors [68], although it is likely that individual factors such as behavioral style also influence the amount of time spent in infant care [69]. Fathers and older male helpers contribute the most, which has been explained as a courtship strategy directed towards the reproductive female [70,71], and female helpers often contribute the least [6], but despite this, they avoid aggression and delay eviction from their group [3]. If the grooming network of the group is adapted to maintain bonds between individuals that cooperate in infant care, it is possible that age and sex are relevant attributes that condition the organization of the network.

The present study offers the first investigation into how the new birth of infants affects the social structure of two groups of cotton-top tamarins of different sizes and compositions, as well as whether infant carrying is associated with a change in individual centrality after the birth. Given the importance of adult helpers in the cooperative care of infants [66,67], we expected that the group with experienced adult helpers would suffer a smaller impact on their group structure. We predicted that the group with adult helpers would show a stable grooming network after the birth, while the group without adult helpers would show changes in the network. We also analyzed the possible effect of sex and age on the network, where individuals that are similar in age and sex groom together more than those with differences in these attributes. Additionally, since infant carrying has important costs [2,3,4], and although fathers and helpers often compete to carry infants [64,65], we predicted that the time spent carrying infants would be correlated with an increase in grooming centrality.

## 2. Materials and Methods

### 2.1. Subjects and Installations

In this study, we reanalyzed part of the behavioral data recorded for other purposes in three family groups of the cotton-top tamarin (*Saguinus oedipus)* colony at the German Primate Center (Göttingen). The data were recorded by the second author during a period of 18 months in the years 1994 and 1995; the results of that study are published elsewhere [3,65]. Each group was formed by a reproductive pair and their offspring. Here, we used the observational data of two of those family groups of cotton-top tamarins (Table 1 and Table 2) housed in continuous full contact (group). We excluded data from groups with smaller sample sizes (<5). Each family was housed in an area of 12 m^2^, with headroom of 2.4 m. Observations occurred between 0900 h and 1200 h. We followed all ethical requirements of the Universidad Autónoma de Madrid, as well as the American Society of Primatologists principles. The data that support the findings of this study are available from the corresponding author upon reasonable request.

### 2.2. Data Collection

For the grooming data, we used one-minute focal sampling twice a day per individual, four days per week during a period of four weeks before and nine weeks after the birth of the infants. We observed each individual for a total of 32 min before and 72 min after the infants’ birth. During these focal observations, every 15 s, we instantaneously recorded grooming performed and received, since this interval produced accurate estimates of the true duration of this category of behavior in the cotton-top tamarin [72].

For the data on infant carrying, all occurrences [73] were recorded continuously during 30 min observation sessions that took place 6–7 days a week. We identified the carrier and recorded the carrying time, observing each group for a total of 32 h. We defined infant carrying as the percentage of time each individual spent carrying an infant [3].

### 2.3. Data Analysis

We created grooming network matrices from directed valued data (asymmetrical and weighted networks). We calculated simple ratio indices [74,75] to represent the edges of each network in order to account for the difference in the observation time. We used UCINET to create the sociograms and to calculate the eigenvector centrality. The eigenvector represents the connectedness, as it is a measure of the number of connected nodes of the focal, weighted by the number of connections of those nodes, and it is interpreted as a measure of the social support of individuals [35].

To study the network stability, we compared the network density, measured as the proportions of all possible binary ties that are connected, before and after the birth for each of the groups using the compare-densities bootstrapping function in UCINET. We also analyzed changes in the network structure before and after the birth of the new infants in each of the groups using a Double Dekker MRQAP Regression [76] in UCINET. The MRQAP Regression predicted the grooming network after the birth, based on a series of networks (grooming before the birth of the new infants, age difference and sex). All analyses were performed on the asymmetrical weighted matrices using 5000 permutations and an alpha value of 0.05.

Lastly, in order to investigate whether infant carrying is associated with an increase in social support in the group, we calculated the difference in the eigenvector before and after the birth for all individuals. We then used permutation-based correlations between the increase in the grooming eigenvector after the birth and infant-carrying time to investigate whether individuals that carry infants more often become more “popular”. Permutation-based correlations were calculated using the “RVAideMemoire” package in R version 4.0.2 using 5000 permutations and an alpha value of 0.05.

## 3. Results

### 3.1. Network Sociograms

**Figure 1:**
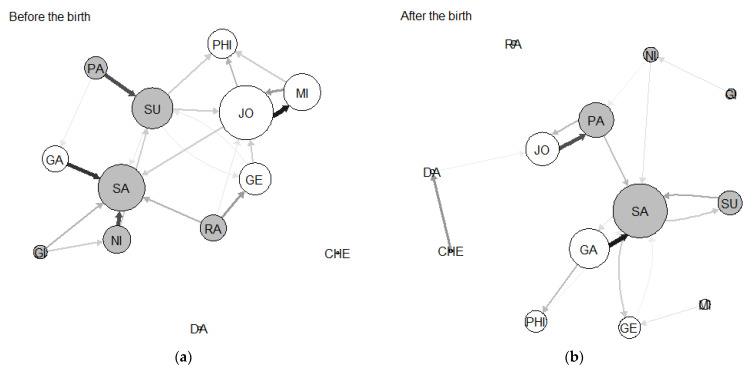
Group A before (**a**) and after (**b**) the birth, displaying ties above the median. The line thickness indicates the strength of the grooming relationship, and the arrowheads indicate the direction. The node size represents the eigenvector centrality, and the node color represents sex: gray nodes are females, and white nodes are males.

The sociogram for group A before the birth showed a bigger group with a total of eight adults, in which most individuals, with the exception of two juveniles, were interconnected with two or more other members of the group (Figure 1). The reproductive female, Sa, showed a highly central position in the group, with a high eigenvector, while the reproductive male, Ga, showed an above-average eigenvector (Table 3). After the birth of the new infants, the group remained well-interconnected, although there were some changes in the position of some individuals (Figure 1). While the reproductive pair remained in similar positions after the birth, two younger females of the group (a subadult, Su, and a juvenile, Ra) saw a decrease in their eigenvectors (Table 3). Data matrices as well as eigenvector scores and infant carrying time are available as File S1.

**Figure 2:**
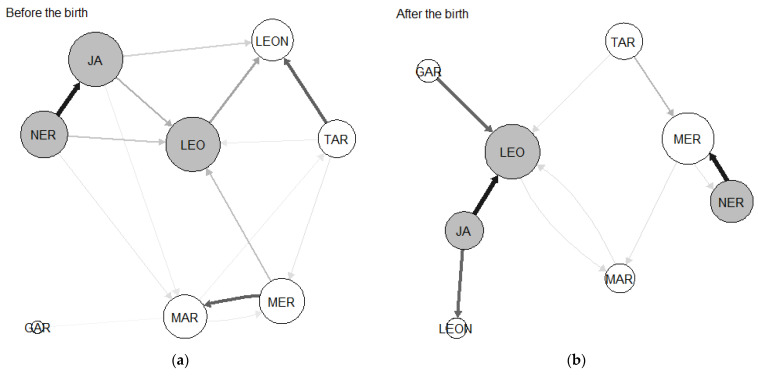
Group B before (**a**) and after (**b**) the birth, displaying ties above the median. The line thickness indicates the strength of the grooming relationship, and the arrowheads indicate the direction. The node size represents the eigenvector centrality, and the node color represents sex: gray nodes are females, and white nodes are males.

In group B, before the birth, the reproductive female Leo was the most central individual in terms of her eigenvector, while the reproductive male Gar had a low eigenvector (Figure 2, Table 4). After the birth, both members of the reproductive pair maintained their positions as the highest and lowest central individuals in the group (Figure 2, Table 4).

### 3.2. Density

There was no significant difference in the density of group A before (density = 13.65) and after (density = 13.85) the birth (*t* = −0.0458, *p* = 0.962). However, we found a significant difference in the density of group B before (density = 47.41) and after (density = 17.14) the birth (*t* = 2.451, *p* = 0.02).

### 3.3. MRQAP Regression

We used MRQAP to investigate the relationship of the grooming network after the birth with the grooming network before the birth and the sex and age of the animals. We found a significant MRQAP model with a moderate effect size for group A (*R*^2^ = 0.196, *p* < 0.001). Grooming after the birth was predicted by grooming before the birth and by age similarity, but not by sex (Table 5).

We found a significant MRQAP model with a small effect size for group B (*R*^2^ = 0.072, *p* = 0.038). None of the predictor variables were significant in the model, although grooming before the birth was close to statistical significance (Table 6).

### 3.4. Permutation-Based Correlations

We used permutation-based correlations to investigate whether the infant-carrying time was associated with an increase in the grooming eigenvector. We did not find significant correlations for group A (*R* = −0.147, *p* = 0.691) or group B (*R* = −0.568, *p* = 0.372).

## 4. Discussion

In this study, we set out to investigate changes in the grooming social network of two cotton-top tamarin groups after the birth of new infants.

The results for the larger group with adult helpers (group A) showed that the grooming network was stable after the birth, and that individuals groomed those of a similar age more often. However, in the case of the smaller group without adult helpers (group B), the analyses did not find the same stability, and there was a lack of effect of age and sex similarity. While group A did not show a change in density after the birth, the density of group B significantly decreased after the birth of the new infants. Additionally, we predicted that the time spent carrying infants would be correlated with an increase in the grooming centrality after the birth. However, the data did not show a significant correlation between the time spent carrying infants and an increase in the eigenvector centrality.

In order to determine whether the group structure was stable, we tested whether the grooming network before the birth could predict the grooming network after the birth. Additionally, we also compared the grooming density before and after the birth to investigate possible changes in the group network. Group A showed a stable group structure, as there was no change in the group density. The results showed a significant regression, indicating that the structure of the network before the birth significantly predicted the structure after the birth. Group B, however, showed a significant change in the network density, which decreased after the birth, indicating a possible disruption of the grooming network after the birth of the new infants. Supporting this change in density, the results for the regression showed that the network before the birth did not predict the structure of the network after the birth of the infants. The discrepancies in these results are likely due to the different age compositions of the groups, as the results also showed that grooming was more common among individuals in the same age group in group A but not in group B. This means that, in group A, adults tended to groom adults more often than they groomed juveniles, and vice versa. However, it is important to keep in mind that it is possible that the smaller sample size in group B affected the results. The smaller sample size carries a lower statistical power, which would explain why the regression model for group B was significant, but the relationship between the grooming network before and after the birth was just above the significance level (*p* = 0.07).

The presence of adult helpers was likely an important factor determining the ability of the group to adapt its structure to the birth of the new offspring: group B was formed by the reproductive pair, two subadults and four juveniles, while group A was formed by the reproductive pair, six other adults, two subadults and three juveniles. The importance of elder siblings in the group increases when they reach adulthood, as they start playing an active role in infant caring [8,72]. This shift in their role is likely associated with a consolidation of their position in the network, resulting in a group structure that is less perturbed by the birth of new offspring. Viewed in this light, our data indicate that groups with adult helpers can better adapt to new members not only because there are more members to provide care [66], but also because the relationships between group members are less likely to change over time. Research into how age affects social relationships in primates is scarce. Evidence indicates that as age advances, the social position of individuals tends to decrease even when their preferred social ties remain stable. Chimpanzees (*Pan troglodytes*) experience a decrease in their social connections as they advance in age, with females becoming less integrated in the group and males reducing their efforts to maintain connections with the overall group while maintaining their roles in smaller cliques [77]. Babary macaques (*Macaca sylvanuus*) experience a similar decrease in their social position, where both males and females display fewer social behaviors as they age [78]. Both studies show that age is an important factor in shaping how individuals position themselves in a group, although both investigated the effects of advanced age in social networks and cannot be extrapolated to how early development affects the social network. This area of research is still underdeveloped, and there is a clear gap in the literature regarding how early development leading to adulthood produces changes in the social position of primates. Future research into how social positions change as a consequence of aging in tamarins will help provide further context to our findings.

Regarding the grooming centrality, the results showed that the time spent carrying infants did not correlate with a change in the eigenvector. This result indicates that individuals that were more involved in infant carrying did not seem to benefit from gaining a more popular position in the group. While this finding does not seem to fit well with an increase in social prestige as a benefit of infant care [52,53], it is likely that grooming centralities reflect complex group dynamics, highlighting the role of grooming as a form of communication and as a tool maintaining social cohesion [25,27,30]. Examining the grooming centrality offered the advantage of producing information about the position of individuals based not only on their relationships with the breeders, but also on their role within the overall group. In this study, we focused on analyzing grooming as the base of social networks rather than looking into other important affiliative behaviors. Behaviors such as infant carrying and food sharing are particularly relevant for cooperatively breeding primates; however, there is evidence that these behaviors are positively linked to grooming between breeders and helpers [36]. This way, grooming is a particularly relevant behavior not only for its own affiliative function, but also because it can be seen as a proxy for other behaviors that indicate strong social ties. In addition to this, grooming offers the advantage of being popular both before and during the presence of infants, which makes it an ideal behavior to study possible structural changes in the affiliative network.

Past studies in cotton-top tamarins [56] and other callitrichids [46,47] focused on examining the relationships between the breeder pair and helpers, rather than studying the grooming network of the group. Interestingly, mothers and fathers are known to groom different helpers based on their contributions to infant carrying: mothers groom helpers that spend more time carrying infants, while fathers groom those that spend the least amount of time carrying infants [56]. This asymmetry in grooming contrasts with that of other species of callitrichids [46,47], where helpers that contribute more often to infant care receive more grooming from both mothers and fathers. This has led authors to suggest that a greater contribution to infant carrying should result in a better standing in the group [36,46]. However, the fact that mothers and fathers seem to have different approaches to grooming helpers [56] could result in a different organization of the grooming network. In regard to our results, examination of the sociograms was helpful in understanding the role of the eldest adult helpers in the network. The two eldest helpers in group A, Phi and Ge, were the highest contributors to infant carrying. Both individuals strengthened their relationships with the reproductive pair in expense of their relationships with other younger members of the group. Interestingly, the eldest helpers of group B, Mar and Mer, did not show a similar strengthening of their relationships with the reproductive pair. In particular, they were not strongly connected to the reproductive male, who was only strongly connected to the reproductive female after the birth. A possible explanation for these findings is that adult helpers have an incentive to strengthen their relationships with the breeding pair to remain in the group, while subadult helpers do not.

While the results of the present study produced interesting findings, it is important to point to some characteristics of the setting and sample that require careful consideration. First, captivity is a common setting for studies of infant care in cotton-top tamarins [2,3,4,8] as well as in other callitrichids [36,46,55]. This setting presents specific challenges that require us to be cautious when interpreting the findings, as captive conditions likely have lower associated costs due to the abundance of easily accessible food and the absence of predation risks, which might decrease the need to actively encourage helpers. Secondly, groups of cotton-top tamarins in the wild range from three to ten members [60], meaning that both of the groups presented in this study had a high number of members compared to what is expected to be found in the field. Taking this into consideration, the importance of the group composition and adult helpers in group A is likely a more robust explanation for the differences found between the groups than a difference in the group size. Nevertheless, the current study shows the importance of carefully considering all social relationships in a group instead of focusing on the breeding pair and presents social network analysis as a useful alternative to study the costs and benefits of cooperative breeding in cotton-top tamarins.

## 5. Conclusions

As a final conclusion, this study is, to our knowledge, the first investigation on how the birth of new infants affects the overall group structure of cotton-top tamarins in captivity. Our findings showed that one group (group A) was stable after the birth and maintained a similar density, while the other group (group B) was not stable and showed a significant change in density. These differences might be explained by the different compositions, as group A was bigger and formed by several adult helpers. Lastly, our findings indicate that a higher involvement in infant care was not correlated with a change in the eigenvector centrality and highlight the usefulness of social network metrics over examining only certain dyadic relationships.

## Figures and Tables

**Table 1:** Cotton-top tamarins of group A (*n* = 13) at the German Primate Center (DPZ) in Göttingen, Germany, 1994.

Name	Age Category	Sex
SA *	Adult	Female
GA *	Adult	Male
GE	Adult	Male
PA	Adult	Female
PHI	Adult	Male
GI	Adult	Female
JO	Adult	Male
NI	Adult	Female
MI	Subadult	Male
SU	Subadult	Female
RA	Juvenile	Female
CHE	Juvenile	Male
DA	Juvenile	Male

Note: adults > 24 months; subadults 13–24 months; juveniles 6–12 months [3]. * Indicates reproductive pair.

**Table 2:** Cotton-top tamarins of group B (*n* = 8) at the German Primate Center (DPZ) in Göttingen, Germany, 1994.

Name	Age Category	Sex
LEO *	Adult	Female
GAR *	Adult	Male
MAR	Subadult	Male
MER	Subadult	Male
NER	Juvenile	Female
JA	Juvenile	Female
LEON	Juvenile	Male
TAR	Juvenile	Male

Note: adults > 24 months; subadults 13–24 months; juveniles 6–12 months [3]. * Indicates reproductive pair.

**Table 3:** Eigenvector scores for group A before and after the birth of the new infants.

Name	Eigenvector Before	Eigenvector After
SA	0.378	0.382
GA	0.282	0.226
GE	0.344	0.345
PA	0.290	0.274
PHI	0.274	0.344
GI	0.191	0.150
JO	0.329	0.419
NI	0.303	0.300
MI	0.138	0.297
SU	0.382	0.226
RA	0.303	0.149
CHE	0.050	0.181
DA	0.058	0.094
Mean	0.256	0.261

**Table 4:** Eigenvector scores for group B before and after the birth of the new infants.

	Eigenvector Before	Eigenvector After
LEO	0.435	0.525
GAR	0.295	0.206
MAR	0.391	0.226
MER	0.391	0.419
NER	0.284	0.382
JA	0.384	0.339
LEON	0.284	0.307
TAR	0.329	0.317
Mean	0.349125	0.340125

**Table 5:** MRQAP regression model predicting grooming after the birth, using grooming before the birth, age similarity and sex similarity as predictors, in group A (*n* = 13).

Variable	Standardized Coefficient	Standard Error	*p*
Grooming before the birth	0.314	0.092	0.003
Age	0.289	6.259	<0.001
Sex	0.087	5.58	0.159

**Table 6:** MRQAP regression model predicting grooming after the birth, using grooming before the birth, age similarity and sex similarity as predictors, in group B (*n* = 8).

Variable	Standardized Coefficient	Standard Error	*p*
Grooming before the birth	0.232	0.062	0.07
Age	0.002	10.428	0.506
Sex	−0.188	9.645	0.109

## Data Availability

Data matrices as well as eigenvector scores and infant carrying time are available as Supplementary Material.

## Supplementary Materials

The following supporting information can be downloaded at: https://www.mdpi.com/article/10.3390/ani13111758/s1, File S1: Data matrices as well as eigenvector scores and infant carrying time.
